# Nest sites as a key resource for population persistence: A case study modelling nest occupancy under forestry practices

**DOI:** 10.1371/journal.pone.0205404

**Published:** 2018-10-11

**Authors:** María V. Jiménez-Franco, Julia Martínez-Fernández, José E. Martínez, Iluminada Pagán, José F. Calvo, Miguel A. Esteve

**Affiliations:** 1 Área de Ecología, Departamento de Biología Aplicada, Universidad Miguel Hernández, Elche, Spain; 2 Departamento de Ecología e Hidrología, Universidad de Murcia, Murcia, Spain; 3 Fundación Nueva Cultura del Agua, Zaragoza, Spain; 4 Bonelli’s Eagle Study and Conservation Group, Murcia, Spain; Austrian Federal Research Centre for Forests BFW, AUSTRIA

## Abstract

Natural nest sites are important breeding resource in terms of population dynamics, especially in forest systems where nest trees limit populations or timber harvesting destroys nests. Nest structures usually have a long life and can be reused by breeding pairs across multiple breeding seasons, so studying their dynamics is of relevance for biodiversity conservation. In this study, we develop a dynamic model to evaluate nest site availability and its influence on the breeding settlement of a forest raptor community composed of booted eagle (*Hieraaetus pennatus*), common buzzard (*Buteo buteo*) and northern goshawk (*Accipiter gentilis*) in a Mediterranean forest ecosystem in southeast Spain. This model approach is also applied to analyse the influence of forestry practices on the dynamics of occupied nests for a simulated period (2010–2050). The simulated scenarios include unmanaged forest and timber harvesting practices of clearcuttings every ten years considering two factors: the age class of trees for clearcutting (40, 50, 60 and 70 years old) and the type of forest management (with or without nest protection). Our simulated results show that the number of breeding pairs is constant during the period without timber harvest, whereas breeding pairs gradually decrease in the scenario of clearcutting trees aged from 70 to 50-years without nest protection, and populations become extinct with the clearcutting of 40-year old trees. Considering the practice of clearcutting and nest protection, nest occupancy can reach the maximum number of occupied nests for the scenarios of cutting 70 and 60-year old trees, and maintain populations without extinction for the scenarios of cutting 40-year old trees. We conclude that nest sites (whether occupied or not) are key resources for increasing the occupancy of the forest raptor community and that nest protection measures buffer the effects of clearcuttings, thus preventing population extinction.

## Introduction

Resource limitation has fundamental ecological consequences for individual performance, population size and community structure [1]. Previous studies have highlighted nest site availability as the main potentially limiting factor of avian breeding populations [1, 2, 3]. Nests constitute key resources for many bird species [4, 5] and reusing them can ultimately influence individual fitness [6]. In several bird species, the same nest can be maintained and reused over long periods of time [7, 8, 9], and interspecific competition may occur when this breeding resource is limited [10]. Indeed, nests are a key resource for population persistence and the conservation of many species, and the availability of nesting platforms and cavities is essential for long-term territorial occupancy [11, 12, 13, 14, 15, 16].

Nest site availability plays an important role for breeding species in forests worldwide and their future conservation status will be highly dependent on forestry policies that avoid the loss of old nest-trees [17]. For example, the removal of trees and snags due to logging is one of the main threats and the cause of the observed decline in many cavity-nesting birds [11, 18]. Forestry practices have altered forested environments and threaten many natural habitats and biodiversity [19]. These practices produce substantial changes in habitat structure through the removal of mature stands that normally provide nesting or roosting sites for animals, while the creation of young homogeneous stands negatively affects several forest species [12, 20, 21]. In harvested forests, the supply of nesting structures can limit the populations of birds and mammals that require cavities and platforms for breeding [17, 22, 23]. Forest raptor species are sensitive to human disturbances and the loss and/or degradation of their original habitat in the face of forestry activities [12, 24]. These species need older trees for breeding [25], so nest sites may be one of their main limiting factors. Therefore, the presence of pre-existing nesting structures may also be an important factor influencing reuse patterns for raptors [4], given that nest building is considered energetically and temporally costly [14]. Several studies have focused on the reuse of nests by forest raptor species, evaluating their occupancy in the subsequent breeding season after logging [26, 27, 28]. However, the effects of forestry practices on biodiversity need to be more thoroughly understood [21, 29, 30], especially the effects of clearcuttings (considering nest protection or the cutting of different age class trees) on the dynamics of occupied nests over a period of years.

Mediterranean regions have been subjected to intensive human disturbances in the past 10,000 years, mainly in the form of forest extractions [31], agricultural development [32] and fires [33]. The effects of forestry treatments (e. g. clearcutting, thinning and pruning) on biodiversity, ecosystem functioning and successional trajectories are poorly understood, particularly in Mediterranean pine plantations [29]. However, several studies have highlighted the fact that many species of forest birds appear to have benefited from forest management practices of moderate intensity [34], or from afforestation and land abandonment [32, 35], although these human-induced disturbances have been detrimental to other animal species [36]. The ability of Mediterranean landscapes to support breeding forest raptors and the effect of different forest management strategies on the maintenance of the bird community have hardly been studied [37].

To provide reliable recommendations for logging procedures compatible with the needs of breeding forest raptors [22, 27, 37, 38], we explored the effect of nest site availability on raptor occupancy in Mediterranean forests using model simulations and a long-term nest dataset. The target raptor species, the booted eagle (*Hieraaetus pennatus*), common buzzard (*Buteo buteo*) and northern goshawk (*Accipiter gentilis*), are long-lived species that show site fidelity and may reuse nesting-platforms over long periods of time [15, 39]; they are also considered sensitive to forest management practices within their nesting areas [38, 40]. The aims of this study were: 1) to determine the influence of nest site availability on the settlement of forest raptors in an unmanaged forest over several years; 2) to predict how timber harvesting practices, more specifically the clearcutting of trees of different age classes (40, 50, 60 and 70-year old, respectively), could affect the raptor occupancy dynamics in the future and 3) to explore the effects of nest protection measures (buffer zones) on raptor occupancy under clearcutting practices. Previous studies have shown that medium and large-sized forest raptors require large nesting trees and mature stands [22, 41] and are vulnerable to modern forestry practices [12, 22, 37], timber harvesting particularly being associated with lower occupancy rates [42, 43, 44]. We hypothesize that forest raptor persistence depends not only on the available habitat in terms of mature forest, but also on the availability of total nests, including non-occupied nests.

To address our objectives, we developed and validated a dynamic model on nest site availability and occupancy of the forest raptor community [4, 15], in order to simulate the long-term dynamics of the following variables under different forest management scenarios: unmanaged forest, clearcuttings of trees of a certain age class and the consideration of nest protection. Model simulations are regarded as an important tool to provide predictions of how avian communities could be affected under different forestry practices and can help guide forest management programs [45, 46]. Therefore, we hope that our dynamic model and the results of simulations might be generalizable to a wide range of taxa and management scenarios with nest site limitations [11, 17].

## Materials and methods

### Ethics statement

Authorization for the study was provided by the Consejería de Agricultura y Agua of the Region of Murcia, which regulates the conservation and management of wildlife and endangered species. Our study, which forms part of a larger investigation into forest raptor populations [4, 15, 39, 47, 48, 49, 50, 51], was observational and did not require invasive techniques. The presence of booted eagle is one of the reasons for which the study areas ‘‘Sierras de Burete, Lavia y Cambrón” and “Sierra Espuña” are designated as Special Protection Areas under Directive 2009/147/EC on the conservation of wild birds.

### Study site and study species

Our study area is located within the Special Protection Area ‘‘Sierras de Burete, Lavia y Cambrón” (ES0000267), located in the centre of the province of Murcia, SE Spain (38°00’ N, 1°45’ W). It is a mountainous area with elevations ranging from 550 to 1,234 m above sea level. The climate is dry Mediterranean, with an annual precipitation of about 400 mm and a mean annual temperature of 17°C. The mountainous landscape is characterized by a forested ecosystem dominated by one tree species, the Aleppo pine (*Pinus halepensis*), a conifer that may reach up to 22 m in Mediterranean areas [52]. Although the study area contains traditional agroecosystems in the valleys (mostly dry-land crops of vine, olive, almond and cereals), the forested areas (13,569 ha) were not substantially disturbed by human activities (such as timber harvesting) during the study period, so most of the forest (about 10,000 ha) can be considered mature [50]. Although this forest suffered a heavy snowfall and strong winds in 2007, where the study species exhibited great tolerance [50], this forest has not suffered any wildfire or relevant diseases, making it a suitable area to study nest dynamics over a period of time. Moreover, the forest area used for validating the dynamic model developed in this study is the Regional Park of Sierra Espuña, a semiarid Mediterranean landscape of c. 20,000 ha located in the centre of the province of Murcia, (SE Spain) (371520N–11340W), which is a Special Protection Area ES0000173 of interest for raptor species [53]. This protected area was reforested during the last years of the XIX and first years of the XX centuries with Aleppo pine [54], under the directive of the national reforestation plan of 1938 [55]. Data on occupied nests in Sierra Espuña come from a historical field study in the area [56] and our own field samples [51].

We studied nesting platforms constructed and used by the three forest raptors in question: the booted eagle, the common buzzard and the northern goshawk. The booted eagle is a trans-Saharan migrant, arriving in the study area in late March and leaving in late September, while common buzzard and northern goshawk are resident in the study area, which represents the southernmost part of their distribution range. The three species have a similar breeding phenology, similar territory sizes (forest area defended by a breeding pair, with nests within less than 300 m from each other) and similar habitat requirements in the study area and are able to exchange nesting-platforms over a long period of time (for more details see Jiménez-Franco et al. [15]).

### Nest location and monitoring

We monitored nests each breeding season, from the end of March to the beginning of May, over a period of 16 years (1998–2013), as part of an intensive long-term monitoring project on forest raptor populations [4, 15, 39, 47, 48, 49]. We assumed that the locations of all nests were known since 1998. We recorded information about the nest building ratio (average annual ratio of nest building per breeding pair) for the study period for the forest raptor community (all species construct nest with similar appearance and dimensions), when a species was observed using a brand-new nest. A nest was considered destroyed when the whole structure had fallen from the nest tree or the branch that sustained it, when the nest tree or nest branches were broken or had fallen, or when most of the nest material (80%) had deteriorated due to natural causes, mainly meteorological perturbations, resulting in a loss of structural integrity [50]. This information allowed us to determine the specific parameter values to be introduced in the dynamic model, such as the amount of mature forest per breeding pair, the nest building ratio and the average nest life expectancy.

We determined a breeding site to be occupied if we observed any sign of territorial or mating behaviour, including territorial flights, courtship, responses to mating calls (e.g. elicited vocalizations, approaches), copulations or by direct evidence of breeding [15]. We recorded variables relating to the characteristics of nest-trees such as nest-tree diameter at breast height (1.3 m above the ground), in order to determine the minimum age of trees for them to be occupied by a breeding pair. We made at least three visits to the nests to observe occupancy and breeding success, so this survey protocol is considered as perfect detection (p = 1) [4, 15].

### The dynamic model NEST

**Fig 1:**
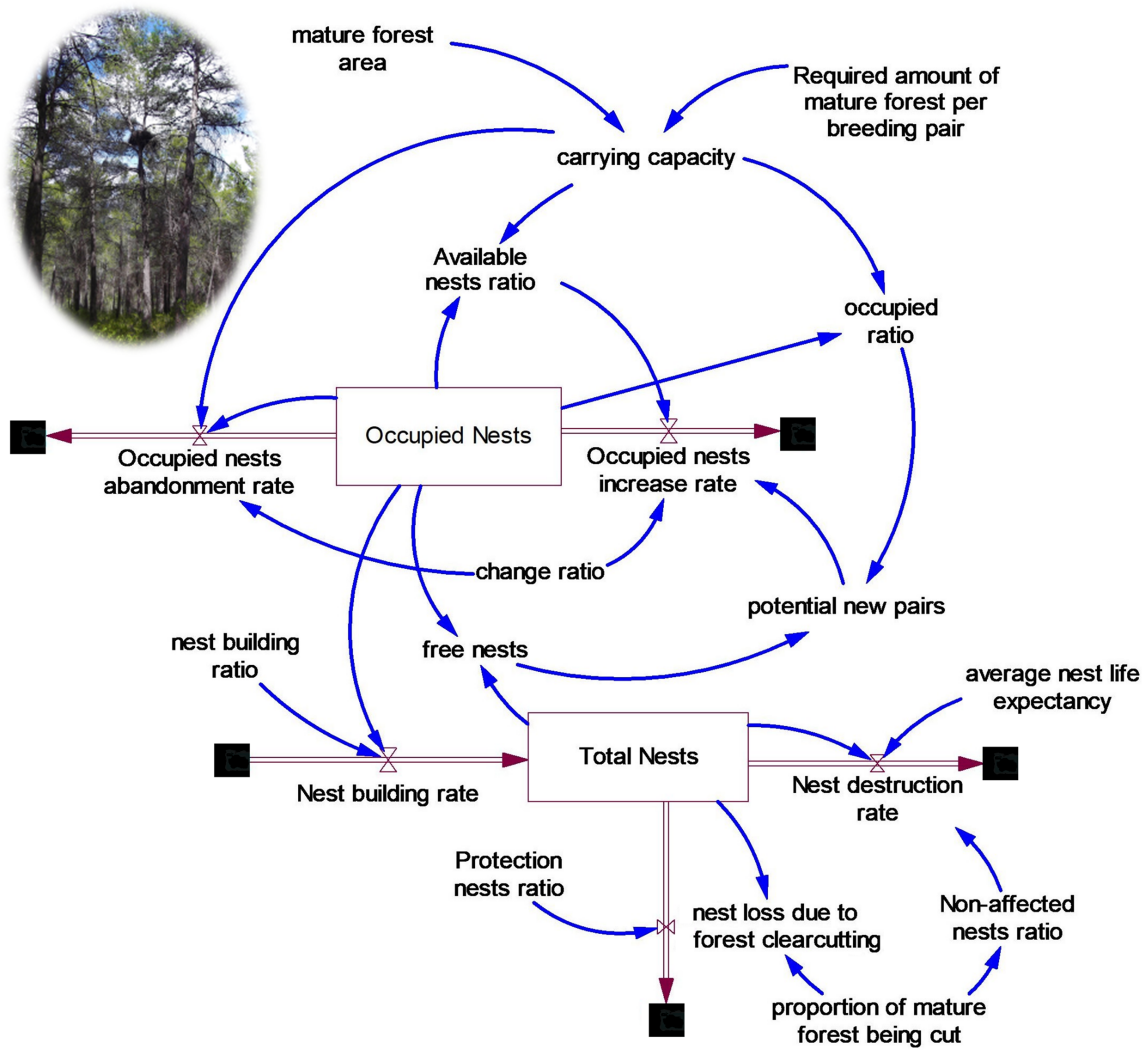
Simplified diagram of NEST. Dynamic model of nest site availability and forest raptor occupancy of the booted eagle, the common buzzard and the northern goshawk in the study area ‘‘Sierras de Burete, Lavia y Cambrón” (SE Spain). The state variables of the system are represented in boxes. The brown arrows represent the rate variables and the blue arrows represent the relationships among model variables and parameters. Note that carrying capacity means the maximum number of occupied nests. Forestry practices are not represented. Picture: forest raptor nest in the study area.

We developed a dynamic model [57], NEST (Fig 1), on the availability of nest sites that can be occupied by members of the forest raptor community, the booted eagle, the common buzzard and the northern goshawk in the Special Protection Area for Birds ‘‘Sierras de Burete, Lavia y Cambrón” (SE Spain).

The model has two state variables: total number of nests in the study area, occupied or not (Total nests, *tn*) and number of nests occupied by one breeding pair of forest raptors (Occupied nests, *oc*). Therefore, occupied nests are equal to the number of breeding pairs.

There are four model parameters: nest building ratio (*nbr*), average nest life expectancy (*nlf*), mature forest area (*mfa*) and amount of mature forest per breeding pair (*mfap*), all of them established by field studies [50]. The parameters nest building ratio *nbr* and average nest life expectancy *nlf* were taken from Jiménez-Franco et al. [15] (see details of parameters in Table 1). No parameters were determined by the calibration of simulation results of state variables against observed data.

**Table 1:** Model parameterisation of the dynamic model NEST, based on empirical data and bibliography on the breeding sites and habitat used by a forest raptor community (booted eagle, common buzzard and northern goshawk) in the study area ‘‘Sierras de Burete, Lavia y Cambrón” (SE Spain) over the period 1998–2013.

Parameter and acronym	Description	Value and units	References
**Nest building ratio (*nbr*)**	Average annual ratio of nest building per breeding pair	0.14/year	Jiménez-Franco et al. [15]
**Average nest life expectancy (*nlf*)**	Longevity of a nest without loss of structure, and so available for occupation	20.68 years	Jiménez-Franco et al. [15]
**Mature forest area (*mfa*)**	Total area of mature forest	10,000 ha	Martínez et al. [50]
**Amount of mature forest per breeding pair (*mfap*)**	Required average amount of mature forest to support a breeding pair	300 ha/pair	Calculated taking into account the total mature forest area of ‘‘Sierras de Burete, Lavia y Cambrón” (10,000 ha) divided by the average number of breeding pairs as suggested by the available data (33 breeding pairs), assuming that the current forest raptor community is at maximum carrying capacity [51].

The maximum number of occupied nests (*moc*; that is, the potential number of breeding pairs) in the study area depends on the area of mature forest (*mfa*) and the area required by a breeding pair (*mfap*) (Eq 1). In the context of this study, mature forest (*mfa*) refers to the trees with an age equal to or greater than the minimum age required for establishing a nest by a breeding pair (34-year old trees), which was determined with the available data on nest-tree diameter and average annual growth of trees [15, 50]. We derived the amount of mature forest area per breeding pair (*mfap*) from the number of breeding pairs in the study area, assuming that this area is at its maximum carrying capacity [51], as suggested by the available data and literature (see details of parameters in Table 1).

The state variable occupied nests (*on*) changes as a function of two opposite effects: (i) the total number of nests (*tn*) (whether occupied or not) and the established number of breeding pairs, which constitute an attractiveness factor which favours the establishment of new breeding pairs (conspecific attraction; [58]); and (ii) the rate of nest occupation decreases when the forest raptor community approaches the maximum number of occupied nests derived from the actual area of mature forest. The rate of nest occupation depends on the maximum number of occupied nests (*moc*), the actual number of occupied nests (*on*) and the total number of nests (*tn*), following Eqs (1–4):

moc=mfa/mfap (1)

onir=anr*or*(tn‑on)*cr (2)

anr=(moc‑on)/moc (3)

or=on/moc (4)

where *mfa* is the mature forest area, *mfap* is the required amount of mature forest per breeding pair, *onir* is the occupied nests increase rate, *anr* is the available nests ratio, *or* is the occupied ratio and *cr* is the change ratio (set to 1/year).

The abandonment rate of occupied nests (*onar*) increases when the mature forest, and therefore the maximum number of occupied nests, is reduced due to forest management (Eq 5):

onar=(on‑moc)*cr (5)

Breeding pairs build new nests at a constant ratio (Eq 6):

nb=on*nbr (6)

where *nb* is the nest building rate, and *nbr* is the nest building ratio.

Nests are destroyed according to the average nest life expectancy (Eq 7):

ndr=tn/nlf (7)

where *ndr* is the nest destruction rate and *nlf* is the average nests life expectancy.

Finally, when the mature forest is cut down, a corresponding proportion of nests is lost (Eq 8):

nlfc=tn*mfcr (8)

where *nlfc* is the nest loss due to forest clearcutting and *mfcr* is the annual proportion of mature forest being cut. The model NEST was developed in VENSIM software [59] and can be consulted in the S1 Appendix.

### Simulation of nest site availability and raptor occupancy in “Sierras de Burete, Lavia y Cambrón”

We simulated the historical process of nest occupancy by the forest raptor community in the study area ‘‘Sierras de Burete, Lavia y Cambrón” from 1890 to 2018, obtaining the model results for the two state variables (total number of nests and occupied nests). This base run simulates the historical dynamics of forest raptor occupation in the study area, starting with the whole area as mature forest (which was the real situation in the study area during the whole study period) and only one breeding pair, in order to represent the raptor occupation dynamics over time.

### Model validation: Simulation of nest site availability and raptor occupancy in “Sierra Espuña”

We tested the model NEST for internal consistency and validated its application with an independent dataset by means of a different forest area, the Regional Park of Sierra Espuña (see details of this natural area in study site and study species section), whose data concerning occupied nest sites in different years were not used to develop the model. Model testing included structural tests, specifically units check or dimensional consistency check, extended simulations, extreme condition tests and a sensitivity analysis [60]. We ran the model for the Regional Park of Sierra Espuña without changes in the model parameters. Changes refer only to the total forest area (higher in Sierra Espuña), and the initial forest age distribution, which considered all trees to be one-year old, since the forest in Sierra Espuña was the result of a reforestation which took place during the first years of the XX century [54]. The simulation time was from 1910 to 2018.

### Simulating the effect of forestry practices

We used the model NEST to determine the following in the study area “Sierras de Burete, Lavia y Cambrón”: 1) the effect of clearcutting trees of different ages on the long-term breeding pairs; 2) the role played by the total number of nests (occupied or not) on such long-term population values under the simulated forest management with and without nest protection. To address the first point, we simulated nest site availability and nest occupation of breeding pairs for an unmanaged forest and for a forest with clearcuttings of trees of a certain age (40, 50, 60 and 70-year old; [20]). A simplified forest submodel accounted for the distribution of forest area according to tree age (one-year age classes from 1 to 70 years) and allowed the cutting of trees of different age classes (40, 50, 60 and 70-year old) every 10 years. The area of mature forest for the raptor community (trees of 34 years old or older) was higher for clearcutting scenarios with older trees [25]. To address the second objective, for each age of cut trees, we compared the maximum number of occupied nests (that is, the potential number of breeding pairs at carrying capacity in Fig 1) that was expected according to the mature forest area existing each year, with the actual number of occupied nests. The difference between both variables expressed the effect of total nests on the occupation dynamics. We considered different forest management scenarios for the period 2010–2050. In all scenarios, we set the initial number of occupied nests to the maximum number obtained from the base run. We then conducted nine scenarios, varying the age of cut trees and nest protection:

- Unmanaged forest: this scenario simulated the nest site dynamics in the study area without forest management practices, therefore, the whole area was considered as mature forest (the real condition in the study area).
- Clearcutting of trees with a certain age without nest protection: these four scenarios were based on modern forestry practices for timber harvesting, consisting of the clearcutting of trees of a certain age [12]. The initial age class distribution of the forest was set to be at long-term equilibrium under each cut cycle (40, 50, 60 and 70-year old). Trees were cut every ten years.
- Clearcutting of trees with a certain age with nest protection: these 4 scenarios simulated nest site dynamics based on clearcutting of trees of a certain age (40, 50, 60 and 70-year old), adding theoretical buffer zones surrounding all raptor nests (whether occupied or not) as a conservation measure of these reproductive structures [22, 61]. We assumed that under this protection measure, nest loss due to the clearcutting activity was reduced by 90% (considering 100% of protected nests to be too optimistic) without changes in the mature forest area.

## Results

### Simulation of nest site availability in “Sierras de Burete, Lavia y Cambrón”

**Fig 2:**
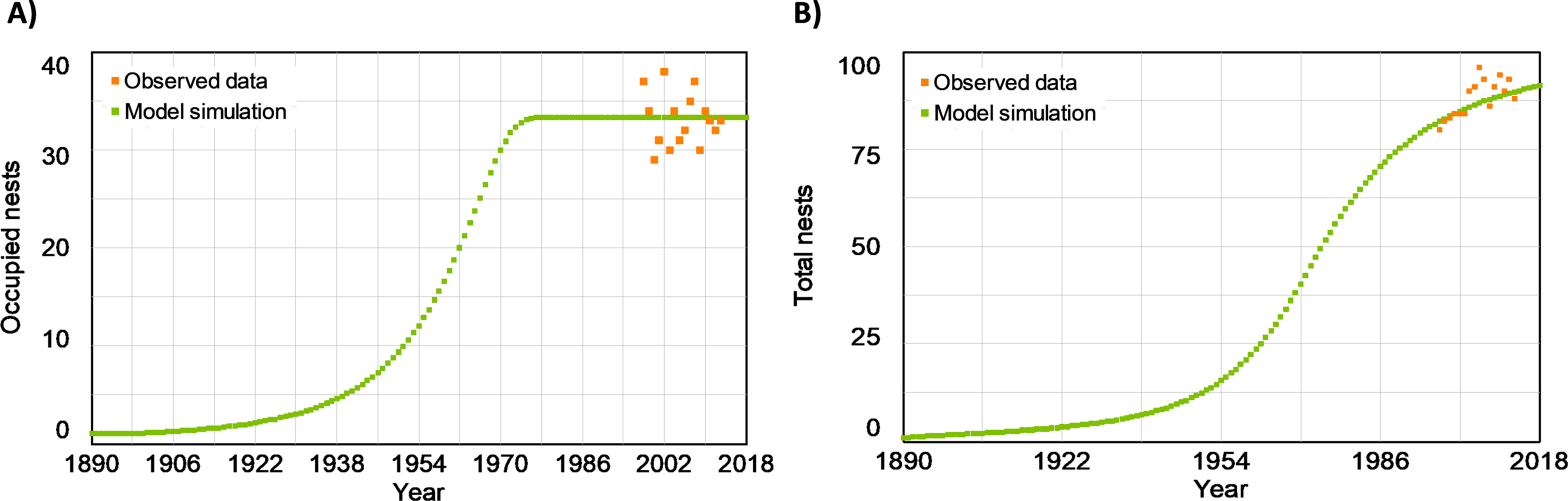
Simulation of nest site availability for the study area “Sierras de Burete, Lavia y Cambrón” (SE Spain) from 1980 to 2018. (A) Nests occupied by the breeding forest raptor community of booted eagle, common buzzard and northern goshawk. Points show the observed nests occupied by the forest raptor community during the observed period (1998–2013) [15]. (B) Total number of nests (nesting-platforms) in the forest area. Points show the observed data of nests for the observed period (1998–2013) [15].

This initial simulation showed the historical process of territorial occupancy by the forest raptor community in “Sierras de Burete, Lavia y Cambrón” until the maximum number of occupied nests was reached (around 33 occupied nests, Fig 2A). The number of total nests increased over time but the rate of increase has slowed in recent decades (Fig 2B). The data observed for both variables during the study period were coherent with the simulation results (Fig 2).

### Model validation: Simulation of nest site availability in “Sierra Espuña”

**Fig 3:**
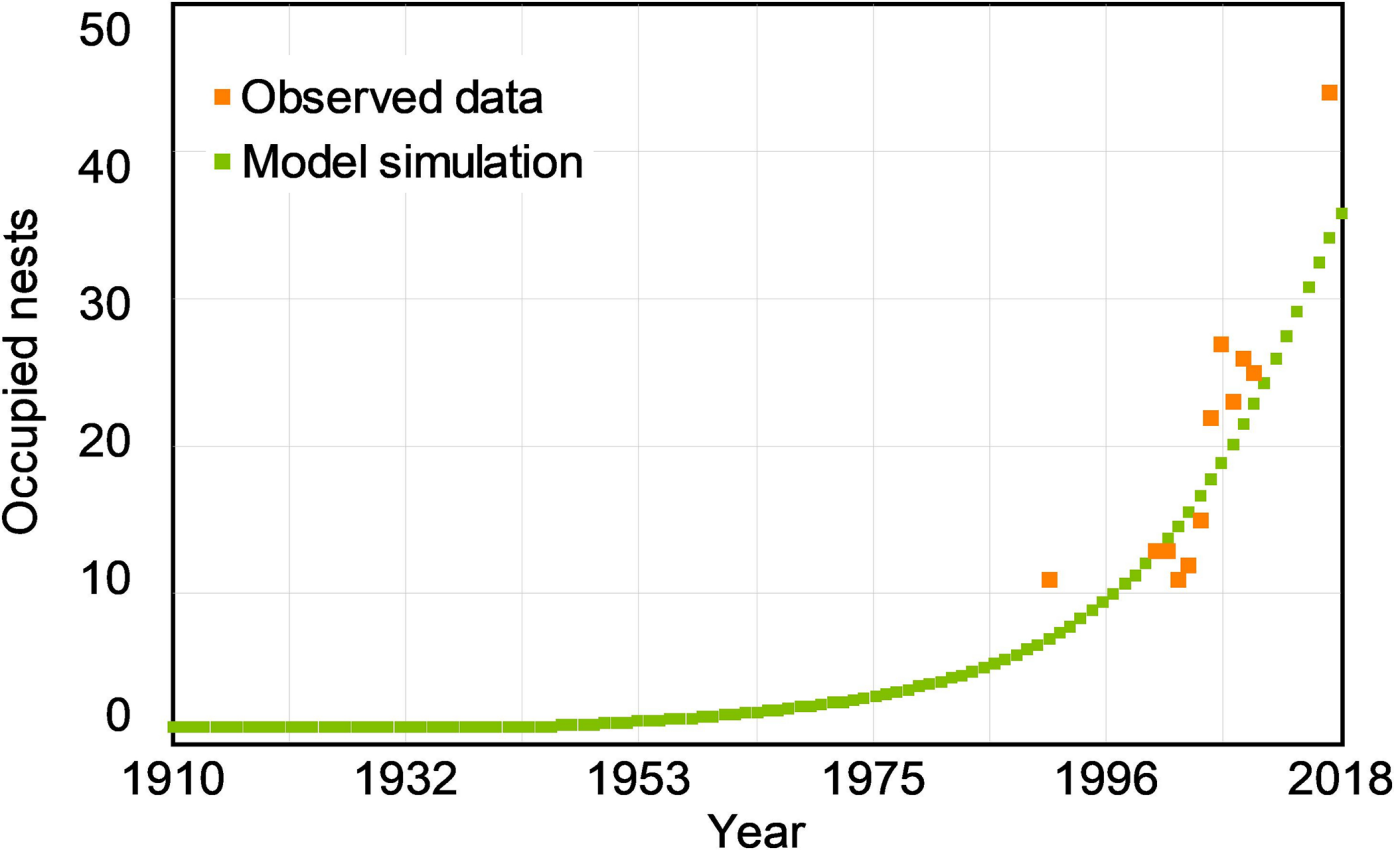
Model validation with an independent dataset. Simulation of occupied nests by breeding pairs of booted eagle, common buzzard and northern goshawk in the Regional Park of Sierra Espuña (SE Spain) from 1910 to 2018. Points show the observed nests occupied by the forest raptor community during the observed period (1991–2017; S1 Table). The data observed in Sierra Espuña was not used for the development of the NEST model or its calibration.

The simulation run corresponding to "Sierra Espuña" showed that there was a rapid increase in the number of occupied nests in the Regional Park of Sierra Espuña from the historical period to the present (Fig 3). These simulated results were consistent with the observed data (S1 Table), although these data were not used to develop or calibrate the model NEST (Fig 3). The model NEST could explain the trend in the number of occupied nests in Sierra Espuña without changing the model parameters. This validation with an independent dataset contributes to our confidence in model’s performance. Moreover, the simulation in Sierra Espuña showed that the model was able to explain the observed data during the transition phase (that is, before reaching the saturation conditions in the raptor community).

### Simulating the effects of forestry practices

#### Effects of clearcutting of trees of different ages

**Fig 4:**
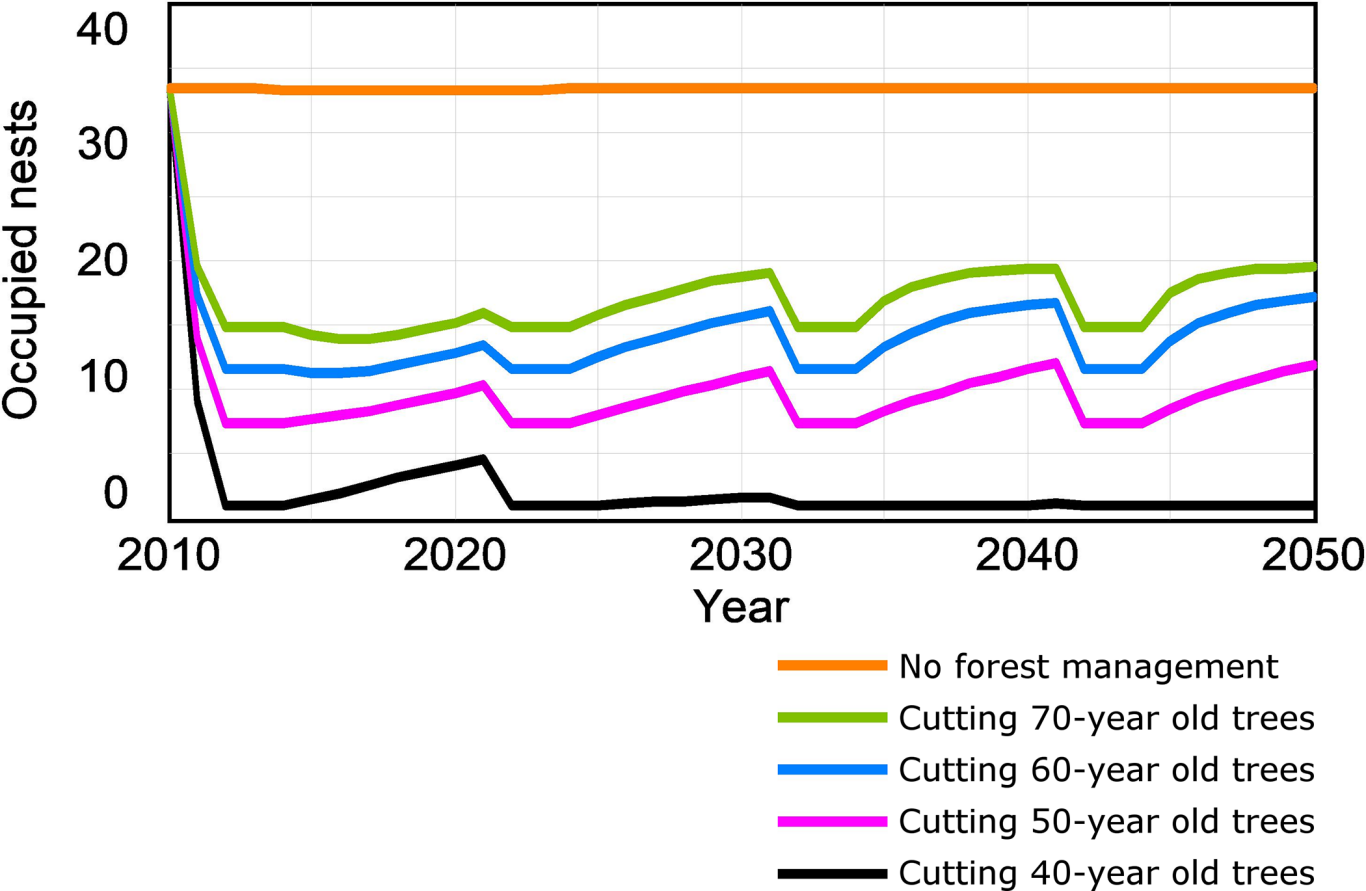
Effects of clearcutting trees with a certain age class. Simulation of the occupied nests of the forest raptor community (booted eagle, common buzzard and northern goshawk) in “Sierras de Burete, Lavia y Cambrón” (SE Spain) from 2010 to 2050, considering five scenarios: one scenario with the absence of forest management and four scenarios with a certain age of cut trees.

The number of occupied nests decreased as the age of trees that were clearcut decreased (Fig 4). Even cutting trees within the highest age class (70-year old) would have a strong effect on the forest raptor community, causing the number of breeding raptors to drop by up to 50% compared with the unmanaged forest scenario. Furthermore, this effect was not linear, but increased when the age of cut trees was reduced. In the scenario where 40-year old trees were cut, the number of occupied nests reached zero and the forest raptor community was not viable (Fig 4).

#### Effect of total nests on the long-term population

**Fig 5:**
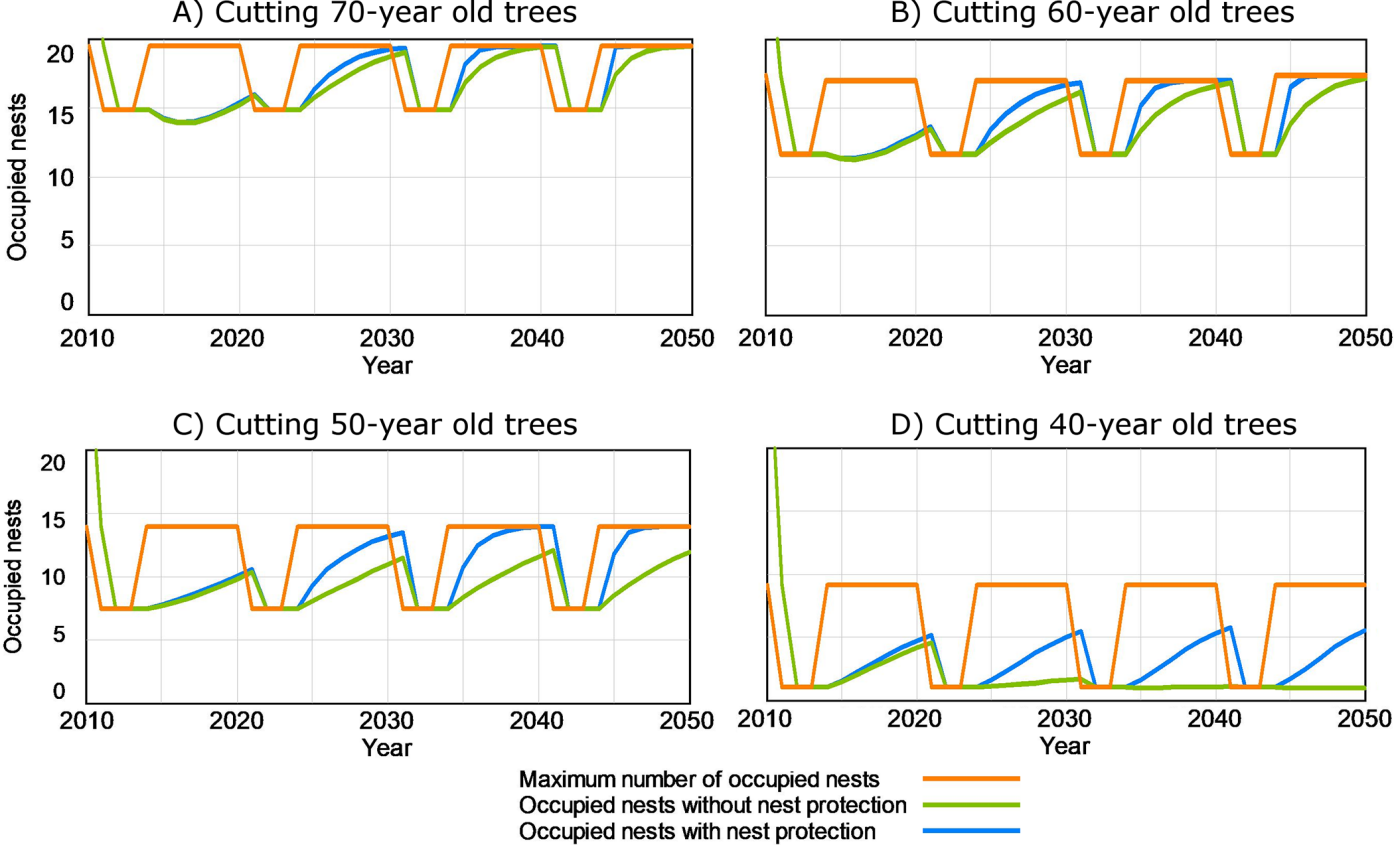
Raptor occupation variables under scenarios with different forestry practices in “Sierras de Burete, Lavia y Cambrón” (SE Spain) during the simulation period (2010–2050). Maximum number of occupied nests according to mature forest existing each year and effective number of nests occupied by breeding pairs of the forest raptor community (booted eagle, common buzzard and northern goshawk) under scenarios with and without a measure of nest protection, and considering clearcutting every 10 years of all trees of a certain age: (A) 70-year old; (B) 60-year old; (C): 50-year old; (D): 40-year old.

**Fig 6:**
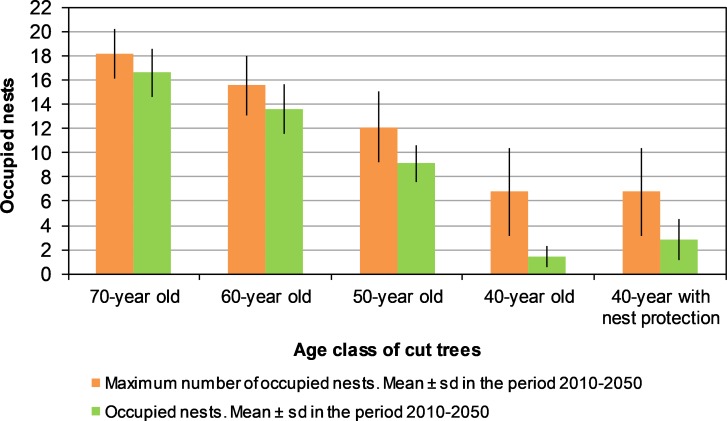
Average values of occupation variables for the forest raptor community (booted eagle, common buzzard and northern goshawk) under scenarios with different forestry practices in “Sierras de Burete, Lavia y Cambrón” (SE Spain) during the simulation period (2010–2050). Average values (mean ± sd during the simulation period) of the maximum number of occupied nests according to the mature forest area existing each year and the effective occupied nests, with clearcutting of trees of a certain age without nest protection. Note that the scenario of cutting 40-year old trees also considers nest protection.

Under each clearcutting scenario, Fig 5 compares the maximum number of occupied nests which might be expected according to the mature forest existing each year with the actual number of occupied nests. The differences between both variables in Fig 5 and also their average values for the whole simulated period (Fig 6) show the role played by the existing number of total nests, whether occupied or not, on the long-term occupancy by the forest raptor community. In the scenario of clearcutting 70-year old trees, the number of occupied nests was below the maximum number of occupied nests, although the number of occupied nests reached the maximum number by the end of the simulation period (Fig 5A). The average for occupied nests in the whole period was only 8% below the maximum number of occupied nests (Fig 6). Under the scenario of clearcutting 60-year old trees, the results were similar, but the difference in the average values between the maximum number and the effective number of occupied nests increased to 12% (Fig 6). When clearcutting 50-year old trees, the maximum number of occupied nests could not be reached during the simulated period (Fig 5C) and the difference between average values of both variables rose to 24.8% (Fig 6). Finally, when clearcutting 40-year old trees, the breeding pair community was not viable, even though a small population of 6–7 pairs might be expected according to the existing area of mature forest (Fig 6). In short, the role of the number of total nests, whether occupied or not, on the raptor occupancy, increased when the age class of cut trees decreased.

#### Effect of nest protection

The effect of protecting all nests (whether occupied or not) during clearcutting (ensuring 90% of nest loss due to clearcutting was avoided) is also shown in Fig 5. In the case of clearcutting 70, 60 and 50-year old trees, the protection of nests shortened the time required to reach the maximum number of occupied nests (Fig 5A–5C, respectively). When 40-year old trees were clearcut, nest protection avoided the local extinction of the breeding pair community, although the maximum number of occupied nests was not reached (Fig 5D).

## Discussion

This study corroborates our previous hypothesis about nest site availability as a critical resource for the territorial occupancy of forest raptors (booted eagle, common buzzard and northern goshawk) in a Mediterranean forest in SE Spain [4, 15]. Our simulations of nest site availability and nest occupancy in a mature and unmanaged forest with the passage of time point to an increase in the total number of nests until a maximum of 33 occupied sites is reached. This result suggests that an unmanaged forest with the presence of old nests could favour breeding pairs of forest raptors, which would reuse nests in successive years [4]. The results show that lowering the age of cut trees has a negative effect on nest site occupancy. Indeed, the scenario of cutting 40-year old trees is nonviable for the persistence of the breeding forest raptor community without nest protection. These results are quite obvious since with as the age of cut trees decreases, the area of mature forest and therefore the available habitat for breeding raptors decrease. However, the most interesting results of this study relate to the simulations of clearcutting, with and without the protection of all nests in the study area, which allow us to understand the important effects of these breeding structures on raptor occupancy. The simulation allowing for nest protection using a forested buffer around known nests shows that the number of nesting raptors depends not only on the available habitat (mature forest according to the age class of trees cut) but also on the total number of nests, whether occupied or not. After a clearcutting, the forest raptor community reaches the maximum number of occupied nests faster when nest protection is considered. This measure of nest protection favours the persistence of the raptor community despite cutting trees of the youngest age class (40-year old). The positive effect of the protection of nests is particularly important when the age class of trees cut is lower.

Our dynamic model of raptor occupancy corroborates the observed patterns of occupied nests in both study areas during the study period, while only the data of “Sierra Espuña” are used for model validation. Although previous studies have simulated the effects of artificial nesting-boxes on population size of kestrels, lag effects on hollows in superb parrots and the protection of bald eagle nests from human disturbance [46, 62, 63], to the best of our knowledge, this is the first ecological application that explores the importance of the availability of nests, including non-occupied nests, in Mediterranean forests and the effects of different forestry practices on the response of forest raptors dynamics over time.

### Breeding sites as a critical resource for population maintenance

Although some studies highlight the importance of nests (e.g., for cavity-nesting species [11]), few studies have highlighted the importance of nesting-platforms in forest raptor species [12, 22]. Although old and unoccupied nests seen to have no apparent function in forest systems, their existence can play an important role in long-term reuse by raptors and may go unnoticed in the usual forest management and biodiversity conservation strategies, where the preservation of non-occupied raptor nests from forest clearcutting is not considered [12, 37]. Forest ecosystems may be strongly influenced by changes in the number of potential nest trees, with the overall effect dependent on stand composition, location and tree size, old stands being especially relevant for the establishment of some bird populations [25]. The results of the present study show that nests are important breeding resources for forest raptors and should be preserved as part of forestry practice. Our conclusions agree with previous studies of other raptor species [16, 62, 64], as well as cavity-nesting species [11, 17, 23, 65]. Moreover, our results corroborate previous studies in our study system that state that all nest trees should be kept in order to preserve an adequate supply of breeding sites for raptors [4]. Although the habitat quality of the study area “Sierras de Burete, Lavia y Cambrón” is similar to that of other mountain ranges in the province of Murcia, breeding pairs do not follow ideal despotic distribution patterns in this province, but an aggregated pattern of territories, with a high density of breeding pairs in the study area [51]. This ecological pattern agrees with previous studies where individuals establishing new territories probably use cues based on location of the information producers (‘location cues’), which may be social cues such as the presence of conspecifics or heterospecifics [4]. Conspecific attraction is a particularly important factor in migratory bird species, where the time of arrival and settlement is short and saves them the effort of searching for territories [66]. Therefore, this pattern of conspecific attraction may be related to the process of individual experience and territorial fidelity in the case of booted eagle [39]. In addition to the importance of the availability of mature forest for raptor occupancy, the results presented in this study provide additional evidence of the critical role of nests.

### Dynamic model as a tool for predicting nest site availability

There is a clear need for more information about the impact of nest site availability on the population size of different species in several ecological contexts [65]. Although some studies evaluate the natural conditions of nest site availability over time [44], it is perhaps more relevant to describe these effects with a before-after control-impact study design, i.e. the BACI method [12, 67]. Experiments that alter nest site availability by providing or removing nest boxes, cavity blocking or creation, or snag removal or creation have provided direct evidence of nest site limitations in some systems [3, 65]. Nevertheless, such long-term studies of the effects of habitat alterations are scarce because management cannot usually wait the years or decades required to study long-lived species such as raptors [37, 62]. In this sense, the design of simulation models, such as our dynamic model NEST, which describes nest site availability, could be a relevant tool for understanding these breeding resources, for modelling future trends and planning forestry policies. However, there are few studies that have simulated nest site availability, probably due to the lack of information on these reproductive structures in relation to breeding species. As far as we know, only two studies have evaluated nest site availability by means of simulation models: McClure et al. [46] examined the usefulness of artificial nest sites to improve the population trends of American kestrels, and Manning et al. [63] examined the effect of different conservation scenarios on potential nest trees for an obligate cavity-nesting species to illustrate the risk associated with lag effects. However, our study is the first that simulates the effects of natural nesting-platforms used by raptors in a Mediterranean forest.

In the present study, a very simple dynamic model was capable of explaining the observed data for nests in a Mediterranean forest in SE Spain, including validation with an independent dataset from another forest area. This model is also capable of offering useful insights regarding plausible forest management practices for forest raptor conservation purposes. Therefore, it has been used to explore the potential effects of different forestry strategies on the dynamic of forest raptor nests. It is important to consider that if management scenarios are excluded, the NEST model has only four parameters (one of its strengths).

Our ecological application is based on forest raptor species breeding on nesting-platforms so our model has been developed for three species which prefer mature forests and have similar patterns of nest occupancy. However, our model could be adapted to raptor species with other habitat preferences, e.g. open landscapes. Moreover, the development of this dynamic model and the results of our simulations are generalizable across a wide range of taxa that depend on nest site availability [11]. Our dynamic model also assumes that clearcutting practices do not affect the habitat structure, only the area of such habitat (i.e., the area of mature forest, according to the age of cut trees). Nevertheless, our model could implement a more realistic spatial configuration of forest in relation to the different types of timber harvesting [25, 61]. Future research lines could also include stochasticity, where potential random changes such as weather effects, wildfires or diseases, could destroy the number of nests in the study area (e.g. by heavy snowfall and strong winds [50]).

### Forestry practices and implications for nest sites

Forest landscapes are one of the most tractable examples of human influence on pristine ecosystems and habitats [30]. The incorporation of the effects of forestry operations in studies of occupancy has recently been studied in North American and Eurasian forests [12, 22, 28, 44], but little is known about the response of sensitive species such as raptors after logging in Mediterranean forests [37]. Therefore, it is crucial to know how bird populations will respond to changes in forest structure in Mediterranean forests [36, 25] and there is a need for greater understanding of the ecological mechanisms that drive these responses [68]. Our simulations suggest that both the maximum number of occupied nests and the effective occupied nests are reduced in a non-proportional way as the age of cut trees decreases. These results agree with the previous hypothesis that timber harvesting limits the available habitat, but also nest site availability and, therefore, forest raptor settlement. Some experimental studies have shown similar results with two of the study species, common buzzard and northern goshawk [12, 44] and other forest bird species [67]. That is, forestry practices such as clearcuttings have negative effects, destroying nests [22, 23] and decreasing biodiversity [67].

When clearcutting activity has intensive effects on the forest structure, special care should be taken regarding timing and methods to ensure population viability. This could involve reducing human activity in areas subjected to intensive forestry operations [37] or performing effective management and conservation strategies based on preserving nests using buffer zones [62]. For this reason, we simulate clearcuttings with theoretical buffer zones as a protection measure [69]. Although the model NEST does not include a variable for buffer areas dimensions in the different scenarios of clearcutting with nest protection, we suggest that these theoretical buffer areas could have 300 m when this measure is applied in the field, according to the minimum distance between nests in the study area [15] and the dimensions considered in experimental studies [46, 61, 70]. Our results show that when cutting trees of the lower age class, it is important to protect all nests, whether they are occupied or not. Previous studies on buffer effects agree with our simulated results, considering nest protection within a buffer zone as a forestry management practice in the case of our study species [12, 71] and other raptors [61, 62, 70].

Other management measures such as the provision of artificial nest sites [72] have been successfully applied in the case of secondary cavity-nesting birds (mostly insectivorous birds and raptors) such as kestrels [16]. However, some birds use dense forests where these artificial structures may not improve populations; for example, these nests may function as ecological traps in the case of forest-dwelling raptors [41]. Therefore, although few studies have evaluated the installation of nest boxes on the population dynamics of birds [46, 63], we suggest that future studies about the application of dynamic models should preferentially focus on other ecological issues that may affect Mediterranean forests, such as climate change or wildfires [73].

In conclusion, some practical lessons for raptor conservation can be derived from the insights provided by our model. Its application provides an early warning approach that conservation managers can use to identify species at risk from longer-term ecological trends so that they can initiate rigorous conservation measures. Without such approaches, there is a considerable risk that some species will find bottlenecks in key resources, such as nest site availability [17].

## Supporting information

S1 Table Number of occupied nests in the Regional Park of Sierra Espuña (SE Spain) from 1991 to 2017, considering the three forest raptor species. (DOC)

S1 Appendix Code of the model NEST in Vensim programme. (DOC)
