# Supplementary material for: Nest sites as a key resource for population persistence: A case study modelling nest occupancy under forestry practices
Source: PLoS One. 2018 Oct 11;13(10):e0205404. doi: 10.1371/journal.pone.0205404 (PMC6181357; doi:10.1371/journal.pone.0205404)
Supplement: S1 Table — (DOC) [file pone.0205404.s001.doc]

Supporting information for

**Nest sites as a key resource for population persistence: a case study modelling nest occupancy under forestry practices**

María V. Jiménez-Franco^1, 2^*, J. Martínez-Fernández^3^, José E. Martínez^2, 4^, I. Pagán^2^, José F. Calvo^2^, Miguel A. Esteve^2^

^1^Departamento de Biología Aplicada, Universidad Miguel Hernández, Elche, Spain

^2^Departamento de Ecología e Hidrología, Universidad de Murcia, Murcia, Spain.

^3^Fundación Nueva Cultura del Agua, Zaragoza, Spain

^4^Bonelli’s Eagle Study and Conservation Group. Murcia, Spain.

**Table S1. Number of occupied nests from 1991 to 2017 in the Regional Park of Sierra Espuña, considering the three forest raptor species (booted eagle, common buzzard and northern goshawk).**

| **Year** | **Occupied nests** |
| --- | --- |
| 1991 | 11 |
| 2001 | 13 |
| 2002 | 13 |
| 2003 | 11 |
| 2004 | 12 |
| 2005 | 15 |
| 2006 | 22 |
| 2007 | 27 |
| 2008 | 23 |
| 2009 | 26 |
| 2010 | 25 |
| 2017 | 44 |
