# Supplementary material for: Nest sites as a key resource for population persistence: A case study modelling nest occupancy under forestry practices
Source: PLoS One. 2018 Oct 11;13(10):e0205404. doi: 10.1371/journal.pone.0205404 (PMC6181357; doi:10.1371/journal.pone.0205404)
Supplement: S1 Appendix — (DOC) [file pone.0205404.s002.doc]

**Supporting information for**

**Nest sites as a key resource for population persistence: a case study modelling nest occupancy under forestry practices**

María V. Jiménez-Franco^1, 2^*, J. Martínez-Fernández^3^, José E. Martínez^2, 4^, I. Pagán^2^, José F. Calvo^2^, Miguel A. Esteve^2^

^1^Departamento de Biología Aplicada, Universidad Miguel Hernández, Elche, Spain

^2^Departamento de Ecología e Hidrología, Universidad de Murcia, Murcia, Spain.

^3^Fundación Nueva Cultura del Agua, Zaragoza, Spain

^4^Bonelli’s Eagle Study and Conservation Group. Murcia, Spain.

**S2 Appendix. . Code of the model NEST in Vensim programme.**

**NEST MODEL**

Available nests ratio = ( carrying capacity - Occupied Nests ) / carrying capacity

Units: Dmnl

(anr). Adimensional ratio accounting for the effect of the carrying capacity on the occupation rate of nests.

average nest life expectancy = 20.68

Units: Year

(nlf) Average lifetime of nests.

carrying capacity = mature forest area / Required amount of mature forest per breeding pair

Units: pair

(cc) Carrying capacity in the given area for the prey birds population.

change ratio = 1

Units: 1/Year

Ratio at which the potential new pairs are converted into actual new pairs.

free nests = Total Nests - Occupied Nests

Units: pair

(fn). Number of non-occupied nests.

mature forest area = area trees >= 34 years old

Units: ha

Total area of mature forest

Nest building rate = Occupied Nests * nest building ratio

Units: pair/Year

(nb) Number of nests built by the prey birds each year

nest building ratio = 0.14

Units: 1/Year

(nbr) Annual ratio of nests building per pair.

Nest destruction rate = ( Total Nests / average nest life expectancy ) * "Non-affected nests ratio"

Units: pair/Year

(nd) Natural rate of destruction of nests

nests loss due to forest clearcutting = Total Nests*proportion of mature forest being cut* Protection nests ratio

Units: pair/Year

(nlfc) Rate of destruction of nests due to the mature forest cut-off.

"Non-affected nests ratio" = ( Total nests ratio - proportion of mature forest being cut ) / Total nests ratio

Units: Dmnl

Proportion of total nests which is not affected by the cut-off

Occupied Nests = INTEG( Occupied nests increase rate - Occupied nests abandonment rate, initial occupied nests)

Units: pair

Number of occupied nests (oc), equal to the actual number of prey bird pairs.

Occupied nests abandonment rate = IF THEN ELSE (Occupied Nests > carrying capacity, change ratio * ( Occupied Nests - carrying capacity ), 0)

Units: pair/Year

(onar) Ocupied nests abandonment rate.

Occupied nests increase rate = IF THEN ELSE ( Available nests ratio > 0, change ratio * potential new pairs * Available nests ratio , 0)

Units: pair/Year

(onir). Annual rate of change in the number of occupied nests

occupied ratio = Occupied Nests / carrying capacity

Units: Dmnl

(or). Proportion of occupied nests respect to carrying capacity of nests.

potential new pairs = free nests * occupied ratio

Units: pair

Number of new pairs which might settle in the area as a function of the existence of free nests and the occupied ratio

proportion of mature forest being cut = mature forest cut rate / mature forest area

Units: 1/Year

(mfcr) Annual proportion of mature forest which is being cut

Protection nests ratio = 1

Units: Dmnl

When the managament measure of protection of nests is activated, the cut only destroys a 10% of the nests that would be destroyed if no protection measures are taken

Required amount of mature forest per breeding pair = 300

Units: ha/pair

Average required amount of mature forest to set up a prey bird pair

Total Nests = INTEG( Nest building rate - nests loss due to forest clearcutting - Nest destruction rate, 1)

Units: pair

Total number of nests (tn, occupied and non-occupied).
